# An integrated, modular approach to data science education in microbiology

**DOI:** 10.1371/journal.pcbi.1008661

**Published:** 2021-02-25

**Authors:** Kimberly A. Dill-McFarland, Stephan G. König, Florent Mazel, David C. Oliver, Lisa M. McEwen, Kris Y. Hong, Steven J. Hallam

**Affiliations:** 1 Department of Microbiology and Immunology, University of British Columbia, Vancouver, British Columbia, Canada; 2 ECOSCOPE, University of British Columbia, Vancouver, British Columbia, Canada; 3 Department of Botany and Biodiversity Centre, University of British Columbia, Vancouver, British Columbia, Canada; 4 School of Health Information Science, Faculty of Human and Social Development, University of Victoria, Victoria, British Columbia, Canada; 5 Graduate Program in Bioinformatics, University of British Columbia, Genome Sciences Centre, Vancouver, British Columbia, Canada; 6 Genome Science and Technology Program, University of British Columbia, Vancouver, British Columbia, Canada; 7 Life Sciences Institute, University of British Columbia, Vancouver, British Columbia, Canada; SIB Swiss Institute of Bioinformatics, SWITZERLAND

## Abstract

We live in an increasingly data-driven world, where high-throughput sequencing and mass spectrometry platforms are transforming biology into an information science. This has shifted major challenges in biological research from data generation and processing to interpretation and knowledge translation. However, postsecondary training in bioinformatics, or more generally data science for life scientists, lags behind current demand. In particular, development of accessible, undergraduate data science curricula has the potential to improve research and learning outcomes as well as better prepare students in the life sciences to thrive in public and private sector careers. Here, we describe the Experiential Data science for Undergraduate Cross-Disciplinary Education (EDUCE) initiative, which aims to progressively build data science competency across several years of integrated practice. Through EDUCE, students complete data science modules integrated into required and elective courses augmented with coordinated cocurricular activities. The EDUCE initiative draws on a community of practice consisting of teaching assistants (TAs), postdocs, instructors, and research faculty from multiple disciplines to overcome several reported barriers to data science for life scientists, including instructor capacity, student prior knowledge, and relevance to discipline-specific problems. Preliminary survey results indicate that even a single module improves student self-reported interest and/or experience in bioinformatics and computer science. Thus, EDUCE provides a flexible and extensible active learning framework for integration of data science curriculum into undergraduate courses and programs across the life sciences.

## Introduction

We live in an increasingly data-driven world, where high-throughput sequencing and mass spectrometry platforms have generated a veritable tsunami of multi-omic information (e.g., DNA, RNA, protein, and metabolite) spanning multiple levels of biological organization [1,2]. For example, over 31 terabases of genomic sequence information are created on average per second, and rates are expected to continue to increase with continued technological improvement across the life sciences [3]. In this light, major challenges in the life sciences are shifting away from data generation and processing to interpretation and knowledge translation resulting in a need for increased training in bioinformatics or more generally, data science.

Despite calls to action as early as 20 years ago [4], there remains a sustained, unmet need for bioinformatics training in the life sciences [5]. In 2015, Horton and Hardin described prevalent challenges and opportunities of this mandate in a special issue of the American Statistician focused on teaching statistics students how to “Think with data” [6]. Beyond reforming curriculum in mathematics and statistics, a meta-analysis of surveys from organizations such as the Global Organisation for Bioinformatics Learning, Education and Training (GOBLET), the European life-sciences Infrastructure for Biological Information (ELIXIR), the United States National Science Foundation (NSF), and the Australia Bioinformatics Resource (EMBL-ABR) found the desire for training to be widespread both in terms of geography and career level [5]. While a number of training formats were applicable, the most common ask for undergraduate students was integrated data science training within current degree programs [5]. Such an integrated program would ensure that all students develop core competency needed for continued studies and career development.

In order to meet this clear and present need for data science education in the life sciences, the University of British Columbia (UBC) has launched a number of initiatives in recent years. These include the Master of Data Science program as well as several specialized undergraduate majors like biotechnology (BIOT) and combined computer science–life science programs. While these programs provide a subset of students with in-depth training, they are inherently self-selecting and limit integrated, inclusive development of core competency across the Faculty of Science [7]. Given that data science training is necessary for life science graduates and specialized degree programs do not attract all students, we posit that such training needs to be incorporated into the fabric of undergraduate coursework such that students are able to progressively develop confidence and skills in an active learning process [8,9].

With this teaching and learning paradigm in mind, the Experiential Data science for Undergraduate Cross-Disciplinary Education (EDUCE) initiative was launched in Fall 2017. The following provides an in-depth description of the EDUCE framework as well as preliminary findings from EDUCE modules implemented in microbiology (MICB) and immunology courses at UBC.

## Experiential Data science for Undergraduate Cross-Disciplinary Education (EDUCE)

EDUCE seeks to develop an extensible, progressive, cross-disciplinary, and collaborative framework to equip undergraduate students in the life sciences with core competency in data science. EDUCE curriculum provides students with the most commonly needed data science skills as defined by recent surveys [5]. Focal learning objectives of the EDUCE teaching and learning framework ask students to learn to

- recognize and define uses of data science;
- explore and manipulate data;
- visualize data in tables and figures; and
- apply and interpret statistical tests.

Sub-objectives are defined using discipline-specific data sets, questions, and software. The combined set of learning objectives are achieved through (1) a modular curriculum plugged into existing courses; (2) coordinated cocurricular activities; and (3) a cross-disciplinary community of practice that brings together teaching teams across multiple training levels. The resulting teaching and learning framework allows students to progressively develop core competency over several years of integrated practice.

### Modular curriculum

The EDUCE framework is modular, providing for flexible integration of data science curriculum within a single course or a series of interconnected courses. Modules are self-contained, adjustable bundles of learning materials that can be delivered as stand-alone classes, recurring instances, e.g., “data science Fridays,” or integrated consecutively in support of capstone projects.

Because EDUCE is modular, it overcomes several of the largest barriers to data science education in the life sciences including instructor capacity, student prior knowledge, and relevance to discipline-specific problems [10]. Specifically, modules are developed and delivered using a community of practice model that empowers instruction across different training levels and disciplines [11]. Teaching assistants (TAs), postdocs, instructors, and research faculty cocreate learning materials and work together across courses to implement EDUCE modules resulting in a zone of proximal development for curriculum development [12]. EDUCE modules are accessible for students from a range of specific majors as they assume no prior knowledge and incorporate all necessary introductory and background materials, including materials unique to that module and review activities from previous modules. Finally, module integration circumvents traditional course creation, i.e., new course codes, thus allowing quicker, more broadly available curriculum deployment within and across different universities.

In addition, modular instruction allows for more timely and direct linkages to discipline-specific content than traditional course structures. For example, students cover the global impacts, thermodynamics, etc. of the nitrogen cycle in their regular course content and then immediately enter an EDUCE module in which they quantify and plot nitrogen species in the ocean using R [13]. This aids student learning in both the discipline and data science as students are able to reference prior knowledge [14] while reflecting on the meaning and application of a nascent skill [15]. Thus, modules leverage student interest in their chosen area of study and maximize the relevancy of data science content used in the learning process.

**Fig 1:**
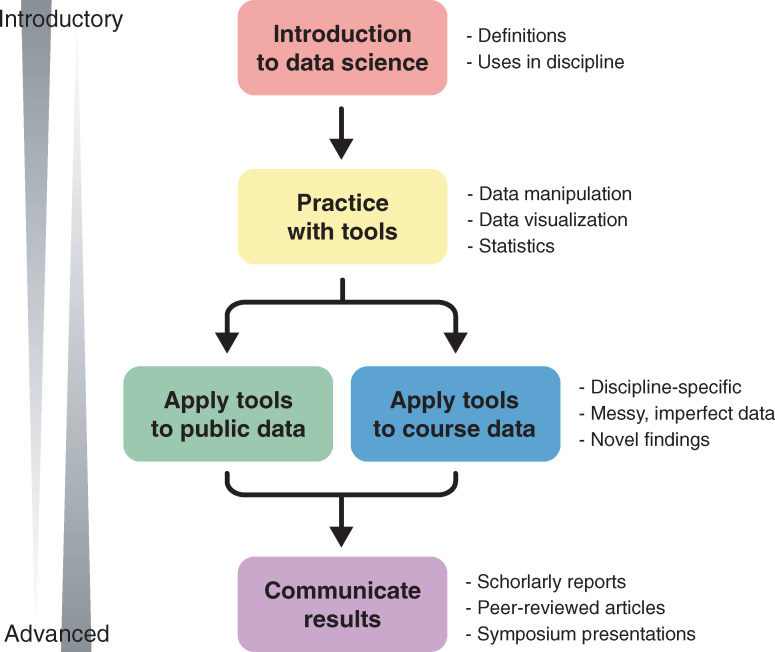
EDUCE module overview. Each EDUCE module consists of introduction, practice, application, and communication. Early, introductory modules are focused on introduction and practice, while later, more advanced modules challenge students to apply their developing data science skills to higher dimensional data with emphasis on visualization, interpretation, and communication. EDUCE, Experiential Data science for Undergraduate Cross-Disciplinary Education.

An EDUCE module consists of all materials related to a set of learning objectives and includes (1) introduction; (2) practice; (3) application; and (4) communication (Fig 1). As students progress from introductory to advanced modules across several courses, the teaching and learning focus transitions from introduction and practice to application and communication. Thus, while each module contains all 4 aspects, later modules build on prior knowledge to both reinforce key concepts and stimulate students to apply developing data science skills to higher dimensional problems with emphasis on visualization, interpretation, and communication. For example, introductory modules use pre-cleaned, tidy data, while advanced modules use raw data requiring numerous tidying steps prior to analysis. Moreover, advanced modules shift toward a research framework with students tackling novel questions with unpublished or under-explored data sets. This affords opportunities for high-dimensional visualization (like multi-panel figures).

This builds progressive competency in data science over several years of instruction without the need for additional course requirements.

Modules vary in size from a single activity to capstone projects, and they can include a range of materials, such as those listed, bundled together based on learning objectives, available class time, and instructor needs. Due to the potentially nonlinear nature of modular instruction, it is important that materials remain accessible to students outside classroom hours and across time as they progress to higher level modules in other courses. For EDUCE, this is accomplished through our public GitHub as well as through efforts to provide fully accessible materials, including:

- self-assessment tools;
- lecture slides;
- notes/handouts;
- in-class tutorials;
- at-home tutorials;
- captioned screen capture videos;
- individual assignments; and
- team projects.

Examples of specific MICB modules are shown in detail below, and additional examples can be found in the “Course Compliler” app on the EDUCE organizational website (educe-ubc.github.io/compiler.html).

### Example modules in microbiology

EDUCE modules have been integrated into 7 third and fourth year MICB courses including lectures, wet labs, and dry labs (i.e., tutorials) (Table 1). Courses were selected based on internal curriculum review, instructor interest, and availability both in terms of the number of hours offered to EDUCE and nonoverlapping time slots with other EDUCE courses. While earlier coursework was considered, and ideally will be part of EDUCE in future, the current program focuses on 300+ level courses able to dedicate at least 3 hours to EDUCE curricula.

**Table 1:** EDUCE courses in MICB.

****MICB code****	****Name****	****L****	****W****	****D****	****Hrs/wk****	****EDUCE hrs****
****301****	Microbial ecophysiology	X			3	5
****322****	Molecular microbiology laboratory	X	X		6	3
****323****	Molecular immunology and virology laboratory	X	X		7	3
****405****	Bioinformatics	X		X	4	17
****425****	Microbial ecological genomics	X			3	17
****421, 447****	Experimental microbiology/molecular biology (CURE)	X	X		7	2+

CURE, course-based undergraduate research experience; D, dry lab; EDUCE, Experiential Data science for Undergraduate Cross-Disciplinary Education; L, lecture; MICB, microbiology; W, wet lab.

**Fig 2:**
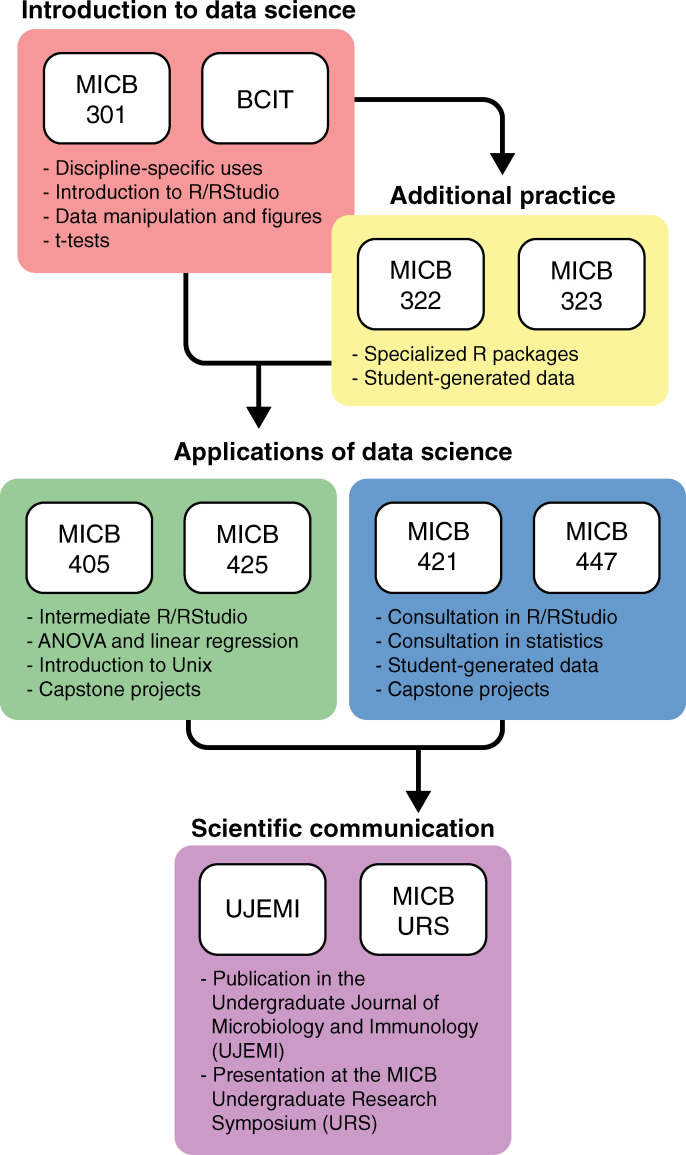
EDUCE curriculum in microbiology. Students enter the program in MICB 301 or equivalent coursework at the BCIT. Some students take additional 300-level courses, MICB 322 and 323. Then, all students progress to 1 or more elective 400-level courses with prescribed outlets for communication of results. BCIT, British Columbia Institute of Technology; EDUCE, Experiential Data science for Undergraduate Cross-Disciplinary Education; MICB, microbiology; UJEMI, Undergraduate Journal of Microbiology and Immunology.

EDUCE content varies in each course and builds from the third (300+) to fourth year (400+) (Fig 2). Ideally, students enter the program in MICB 301 where they master basic R/RStudio scripting to manipulate data, create figures, and perform *t* tests. Then, students acquire additional practice in R/RStudio in 300-level labs (MICB 322 and 323) where they employ specialized R packages to manipulate and plot data generated in the course. For example, MICB 322 uses the Sushi package [16] to visualize transposon insertions in the *Caulobacter* mutant library created by students in the course. Next, students progress to elective 400-level courses including MICB 405 and 425, as well as course-based undergraduate research experiences (CUREs) including MICB 421 and 447. In the elective courses, EDUCE modules are built into the curriculum and include capstone projects using published but under-explored metagenomic and metatranscriptomic data sets [17]. In contrast, CUREs involve student-led research projects that generate novel data. Thus, EDUCE serves as a consultation resource to help students with R/RStudio packages, Unix tools, or statistical methods as required for their individual projects. All EDUCE elective courses offer the opportunity for students to communicate their scientific findings through publication in the Undergraduate Journal of Microbiology and Immunology (UJEMI; jemi.microbiology.ubc.ca) and/or presentation at the MICB Undergraduate Research Symposium (URS; jemi.microbiology.ubc.ca/UndergraduateResearchSymposium).

While the above is an ideal module progression, the EDUCE framework is intentionally adjustable. This is necessary, because MICB prerequisite structure and major requirements do not resolve a linear progression for all students. Microbiology Honours (MBIM) and MICB degrees require the majority of EDUCE courses, while combined majors require a subset of EDUCE courses with little overlap between elective options. Specifically, the MICB/MBIM and Computer Science (+CPSC) or Earth, Ocean, and Atmospheric Sciences (+EOAS) majors require the first EDUCE course (MICB 301) but different 400-level elective courses (MICB 405 and 425, respectively). MICB/MBIM + EOAS also requires an additional 300-level EDUCE “practice” course. The Biotechnology (+BIOT) degree, on the other hand, is a joint program wherein students spend their second and third years at BCIT. Thus, +BIOT does not require any 300-level EDUCE courses. In addition, many students choose to take additional 400-level elective courses beyond those required by their major (Table 2).

**Table 2:** EDUCE course requirements for microbiology majors.

	****Undergraduate major****			
****MICB code****	****MICB/MBIM****	****+CPSC****	****+EOAS****	****+BIOT****
****301****	R	R	R	S
****322****	R	S	R	O
****323****	R	S	O	O
****405****	O	R	S	R
****425****	O	O	R	S
****421/447****	R	O	O	S

BIOT, Biotechnology; CPCS, Computer Science; EDUCE, Experiential Data science for Undergraduate Cross-Disciplinary Education; EOAS, Earth, Ocean, and Atmospheric Sciences; MBIM, Microbiology Honours; MICB, Microbiology; O, optional; R, required; S, suggested.

These course modules incorporate the 4 focal learning objectives and form the basis of the EDUCE teaching and learning framework. While all MICB students obtain some level of data science competency through EDUCE modules within required courses, not all students are exposed equally due to course dependency relationships or specialized program requirements. To help overcome this variation, a persistent cocurricular layer was developed that is coordinated with EDUCE module deployment.

### Cocurricular activities

EDUCE cocurricular activities reinforce and expand on course modules by providing undergraduate students with multiple data science learning opportunities outside of the classroom setting. Similar to course modules, cocurricular activities are flexible and integrated to provide students with progressive levels of instruction from introductory to advanced (Fig 1). Such activities include workshops, hackathons, and directed studies that incorporate 1 or more focal learning objectives and meet the following criteria. They must first and foremost be accessible in terms of cost, content, and schedule. Specifically, undergraduate students are often unable to pay the high costs of data “boot camps” [18] and resist outside training opportunities due to motivational barriers or limited time [19]. Secondarily, EDUCE cocurricular activities directly reiterate and build on learning objectives from related course modules. This feature is intended to improve student learning by referencing prior knowledge [14] and imparting discipline-specific meaning to developing skills [15]. This is particularly important for short-term training opportunities as recent research suggests that fully stand-alone programs, such as “boot camps,” do not promote long-term skill development even at the graduate level [20,21]. Finally, cocurricular activities invoke the same or similar data sets and packages as course modules.

Course modules are coordinated in time with cocurricular activities to prime student participation. For example, in the Fall term, introductory R workshops are offered at the same time that students are introduced to R scripting in MICB301 and provide a refresher for students in MICB405 prior to learning more advanced functions and faceted data visualization. While integration with classroom modules requires that cocurricular activities are discipline-specific, this does not necessarily exclude other participants. For example, an R [13] workshop using oceanic oxygen data may be designed for life or earth science students but is no less accessible to other disciplines than the commonly used R “cars” data [22]. Regardless of the data set, cocurricular activities teach foundational skills that translate across disciplines, including tidying tabular data, effective data visualization, and troubleshooting with specific (e.g., R “help” function) and more general tools (e.g., Stack Overflow). Thus, while current EDUCE workshops focus on applying data science skills in ecology and microbiology, thematic workshop can be readily developed to focus on other discipline-specific data sets or software applications.

EDUCE cocurricular activities fulfill a number of roles. Workshops provide a relaxed and open environment in which to practice current course modules or review previous ones and allow students opportunities to explore learning modules from courses that conflict with their registered schedules. Workshops also provide the teaching team an opportunity to benchmark new curriculum prior to module integration into classroom settings. Other cocurricular activities such as hackathons provide opportunities for students to work in a more social team setting to solve interesting problems with real-world implications, while directed studies provide students with the opportunity to use their data science skills to answer specific research questions. During workshops and hackathons, substantial time is dedicated to promoting scaffolding between participants across different training levels.

### Example cocurricular workshops in microbiology

The EDUCE program at UBC has partnered with the Ecosystem Services, Commercialization Platforms and Entrepreneurship (ECOSCOPE; ecoscope.ubc.ca) training program and the Applied Statistics and Data Science Group (ASDa; asda.stat.ubc.ca) to deliver data science workshops accessible to participants across different training levels from undergraduate students to industry professionals. The current workshop portfolio (github.com/EDUCE-UBC/workshops_access) consists of 32 hours of content in R [13] including the same oceanic geochemical data set [23] used in most EDUCE course modules. These workshops incorporate the 4 focal learning objectives and include the following:

- introduction to R;
- the R tidyverse;
- reproducible research in R and Git;
- intermediate R programming;
- statistical models in R; and
- phylogenetics and microbiomes in R.

Introduction to R is a short refresher of the MICB 301 module or an introduction to the workshop series for participants not enrolled in EDUCE courses. The R tidyverse closely follows its respective module in MICB 405 and 425 (Fig 1) and thus, can be used as a refresher or additional practice. The remaining workshops have some overlap with course modules (like ANOVA in statistical models in R and MICB 405) but mainly build on the fourth year modules to challenge students to continue developing their data science skills. In 2020, 2 advanced workshops were added including “Introduction to programming and plotting in Python” and “Visualization of metagenomic and metatranscriptomic data.”

Importantly, ECOSCOPE sponsorship allows undergraduates to take any workshop for 10 CAD as compared to regular registration fees of 100 CAD or more. This, along with careful scheduling to (1) align workshop content with undergraduate course modules and to (2) accommodate the most common undergraduate schedules in MICB, facilitates increased undergraduate participation. Workshops are actively advertised in undergraduate MICB courses, the ECOSCOPE website, and multiple campus list serves. If scheduling conflicts prevent a significant number of undergraduates from participating, additional workshop sessions are planned on an ad hoc basis. While current workshops run on a subsidized cost recovery basis, this model can only be maintained with sustained institutional support that ideally would enable registered students to participate for free.

### Community of practice

**Fig 3:**
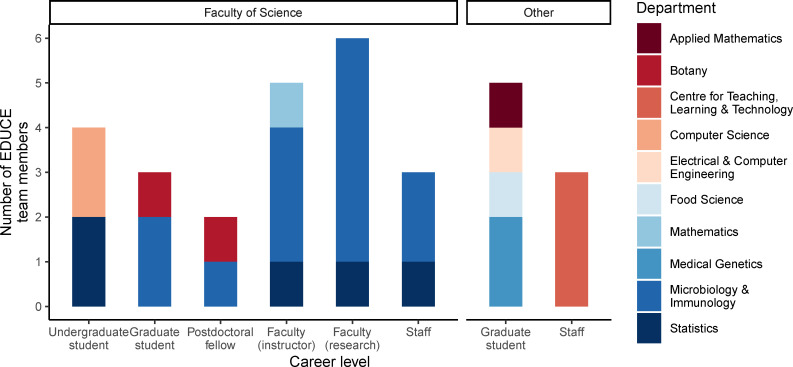
EDUCE team members at UBC. The EDUCE initiative is led by MICB in the Faculty of Science. However, TAs, consultants, collaborators, and other support come from across the campus community and include 10 departments from 3 faculties (Science, Applied Science, and Medicine). Team members include multiple training levels that work together to develop and deploy content in both courses and cocurricular activities. EDUCE, Experiential Data science for Undergraduate Cross-Disciplinary Education; MICB, microbiology; TA, teaching assistant; UBC, University of British Columbia.

Data science is an inherently cross-disciplinary field traditionally comprised of statistics, mathematics, and computer science. EDUCE builds on this tradition through a cross-disciplinary community of practice including traditional and less conventional fields. While EDUCE is coordinated by a research faculty member and postdoctoral teaching and learning fellow (TLF) appointed through MICB, the teaching team spans 10 departments across 3 faculties (Fig 3). For example, faculty in statistics consult on module development, the mathematics department provides resource support, and TAs have been recruited from the Faculties of Science, Applied Science, and Medicine. The community consists of multiple training levels including undergraduate and graduate students, postdoctoral fellows, instructors, and research faculty. Overall, the EDUCE teaching team brings together expertise from across the university to provide relevant curriculum grounded in both current data science best practices and scholarship of education leadership.

The EDUCE community of practice reflects widespread changes in teaching in MICB. A growing number of MICB courses (+24% from 2018 to 2019) are taught by a team of faculty often including both tenure-track researchers and instructors. This team teaching brings additional expertise, styles, and perspectives into the classroom [24,25] and encourages collaboration and community [25]. In addition to faculty, EDUCE TAs contribute to team teaching. Unlike traditional appointments, EDUCE TAs are not assigned to a specific course nor do they always come from the same department. Instead, EDUCE TAs are recruited from across the campus community and participate at every level, from curriculum development to office hours and instruction. This provides undergraduate students in EDUCE courses with diverse resources, viewpoints, and role models as they progress through modules and cocurricular activities. At the same time, EDUCE TAs gain hands-on teaching experience and evaluation to build their individual teaching portfolios.

### Preliminary outcomes of EDUCE

EDUCE launched at UBC in Fall Term 1 of the 2017 to 2018 academic year. At present, approximately 475 (redundant) students are exposed to EDUCE modules through 45 total classroom hours in 5 MICB courses per year. Over the first 2 years, cocurricular workshops were taken by 77 EDUCE students, which resulted in increasing undergraduate participation from 0% (2016 to 2017, prior to partnership with EDUCE) to 8.8% (2017 to 2018) to 23% (2018 to 2019) of total registrants. Undergraduate participants in workshops appears to be stabilizing around 20% in 2019 to 2020. While the majority of undergraduate workshop participants come from EDUCE courses, and thus, are pursuing a degree in microbiology, these cocurricular activities also engaged with students in chemistry, mathematics, economics, and others.

Evaluation of EDUCE is ongoing and includes student surveys with the UBC Behavioral Research Ethics Board approval (BREB H17-02416) at the start (pre) and end (post) of courses (S1 Text) as well as at the end of workshops. Students provided informed written consent and in courses, received 1% extra credit for completion of surveys, regardless of participation in the study. Courses with <5 EDUCE hours (Table 1) were excluded to avoid redundant surveying of students. For example, >95% of students in MICB 322 concurrently take MICB 301. At present, 439 pre-course responses and 400 post-course responses have been collected with 91 students indicating willingness to complete postgraduation surveys or focus groups. An additional 44 responses have been collected from cocurricular workshops.

**Fig 4:**
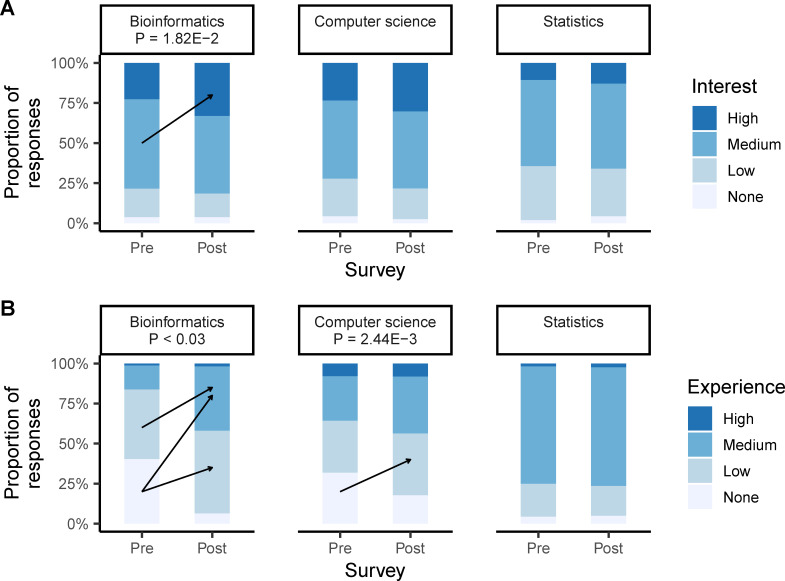
Student interest and experience in data science. Student responses from matched pre- and post-surveys in MICB 301 indicating self-reported (A) interest (*N* = 143–146) and (B) experience (*N* = 136–143) in data science areas including bioinformatics and computer science. Due to changes in survey format, numerical responses from 2018 to 2019 were converted to categorical groups from 2017 to 2018 as none (0), low (1–3), medium (4–7), and high (8–10). Arrows and *p*-values indicate significant response changes by Monte Carlo symmetry tests for paired contingency tables. MICB, microbiology.

Preliminary analysis of the first EDUCE course, MICB 301, from 2017 to 2018 revealed that student self-reported interest in “bioinformatics” increased with a significant transition from medium to high interest (Monte Carlo false discovery rate [FDR] P = 1.82E-2, Fig 4). Similarly, student self-reported experience in “bioinformatics” significantly increased from none to low (FDR P = 1.84E-7) or medium (FDR P = 2.74E-5) as well as from low to medium (FDR P = 2.69E-2). This is relevant to attracting more students to fourth year elective courses implementing EDUCE modules including MICB405 and MICB425. In contrast, self-reported interest in “computer science” remained constant but showed significant gains in experience from none to low (FDR P = 2.44E-3), and “statistics” had no significant changes in either interest or experience. Thus, it appears that even minimal exposure to data science in disciplinary coursework (5 hours, Table 1) may impact student interest and confidence in some data science areas. More importantly, at least 1 case was observed in which a group of students successfully transferred and implemented these skills for their undergraduate research project resulting in a UJEMI+ publication (https://jemi.microbiology.ubc.ca/node/188).

Full analysis details are available at https://github.com/kdillmcfarland/EDUCE_desc.

## Recommendations for implementation

Modular instruction has the potential to impact large numbers of students with minimal disruption to existing university structures. However, there are still some challenges in beginning such a program. Here, we provide recommendations for implementing a new modular data science program based on our experiences with EDUCE in microbiology.

### Personnel

EDUCE functions from a collaborative community of practice with a dedicated lead postdoc. We strongly recommend programs be led by a full-time individual as this person is responsible for overall curriculum flow and coordination as well as for teaching and organizing the material. While EDUCE course modules are designed to be extensible, some modification will be required prior to implementation in a new course to accommodate individual teaching style, course schedule, and student prior knowledge. This process is made easier through collaboration across a community of practice and the use of open-source tools like GitHub. However, as with any new curriculum, instructor preparation time remains a barrier to implementation. A dedicated modular curriculum lead reduces this barrier by removing the need for primary course instructors to develop or teach data science material with which they are unfamiliar. This person also serves as the connection point and main communicator between faculty, TAs, students, and other members of a diverse community of practice.

Currently, EDUCE is led by a postdoctoral teaching fellow, but this position could be equally filled by new or existing faculty or even a graduate student with focus in education. Ideally, modular curriculum has a permanent lead to ensure continuity and keep modules updated with current software and methods. While a dedicated lead may no longer be necessary once a program is somewhat mature, this requires that primary course instructors take over teaching of their course’s module(s) and increases the chances of disjointed or outdated curriculum in later years.

EDUCE also has at least 2 TAs in addition to any TAs assigned to individual courses. These EDUCE TAs greatly add to the knowledge base of our community of practice as they are recruited from outside departments like computer science and statistics, whereas course TAs almost always come from within microbiology. However, if sufficient hours are available from current course TAs, these additional positions are not required, and course TAs could play a larger role in modular instruction.

In addition, modular curriculum is such that a large, department-wide effort is not the only way to integrate data science into current undergraduate programs. Even a single module has the potential to impact student learning and motivate further education. We encourage faculty with an interest in incorporating data science to use our freely available content on GitHub, adapt it as they see fit, and re-share it. This will build a library of open-source curriculum, thereby reducing development time and facilitating incorporation by others. Moreover, institutions with some funds to put toward data science initiatives may consider employing TAs to teach developed modules. Oftentimes, graduate students have experience with the latest software and methods as part of their graduate research. A TA-led modular curriculum leverages this knowledge and provides students with hands-on teaching experience.

### Course selection

EDUCE courses were chosen based on instructor interest, available hours, and common paths to degrees in microbiology. We took a realist approach to where and when modules could be incorporated and then determined what curriculum could be covered within that structure. This was done by (1) identifying our target students (microbiology); (2) mapping the most common paths to degree for these students (MICB, +EOAS, etc.); (3) selecting progressive 300- and 400-level courses that most to all students take; and (4) approaching and negotiating with course instructors for proposed module hours. This process is highly flexible and provides a framework from which curriculum stems.

For future programs, we recommend starting with earlier modules such that 300-level students are able to more quickly progress into discipline-specific applications. These early modules should be small and diverse so that students become familiar with a variety of tools and build confidence in data science. Unfortunately, this was outside the scope of EDUCE in its first years but remains an active area of development.

### Curriculum development

Within our 2-year structure, we then developed curriculum around our 4 focal learning objectives: (1) introduction; (2) practice; (3) application; and (4) communication (see introduction to EDUCE for details). We drew from our community of practice at all steps and advocate that new programs begin partnerships with faculty in statistics, mathematics, and computer science as early as possible.

For undergraduate data science education, the specific tool used is less important than the students’ exposure to data science and practice coding or scripting. EDUCE mainly employs R; however, there exist many tools with similar functionalities. Thus, we recommend any tool that is (1) free and open source; (2) commonly used in the discipline; and (3) familiar to the teaching term. This could be Unix, R, python, Perl, Julia, etc.

Progressive modules also allow for progressive deployment of curriculum. We recommend starting with modules at a single course level and then adding the next levels as those students progress through their degrees. This avoids the challenges we faced in the first year with 400-level courses needing to cover both their own and previous introductory modules. Additionally, progressive deployment lessens the initial development time needed and may aid programs in getting started.

### Course materials

All EDUCE teaching and learning materials are available on GitHub (https://educe-ubc.github.io) with the intention of developing an open-source community of practice that extends beyond UBC. In the future, collaborative platforms like GitHub classroom could facilitate collaboration and material exchange between instructors and strengthen the community of practice within and between universities. Thus, future EDUCE-like programs will not have to start from scratch and can pull from programs like EDUCE, UBC Master of Data Science (https://github.com/UBC-MDS), and UC Berkeley Foundations of Data Science (https://github.com/data-8), to name a few that we found helpful.

## Conclusions

The EDUCE initiative at UBC was launched in Fall 2017 in response to a clear and present need to expand data science education in the life sciences. The initiative seeks to develop an extensible, progressive, cross-disciplinary, and collaborative framework to equip undergraduate students in the life sciences with basic competency in data science. EDUCE learning objectives are achieved through (1) a modular curriculum plugged into existing courses; (2) coordinated cocurricular activities; and (3) a cross-disciplinary community of practice that brings together teaching teams across multiple training levels. The resulting framework allows students to develop progressive data science competency over several years of integrated practice. Initial implementation of EDUCE learning modules and cocurricular activities is focused on ecology and microbiology but can be readily extended to other data sets and other discipline-specific software.

Although preliminary results indicate that EDUCE is having positive impacts on student interest and learning in data science, further analyses of collected survey data are needed to determine if (1) these perceived changes translate into long-term skill development [20]; and if (2) EDUCE curriculum is truly causative of these outcomes. Despite the need for continued data collection to measure EDUCE impacts, the EDUCE framework provides a viable approach to data science education in the life sciences that is both flexible and scalable across in-person and remote learning platforms. As with any other ability, students need the time and scaffolding to learn data science tools, and the relevant education should not be confined to a single course. The EDUCE community of practice works to transcend siloed educational norms resulting in a zone of proximal development that supports both student and instructor achievement. Given that data science competency, also referred to as data literacy, is increasingly recognized as a critical but limiting ability in the modern workforce, institutions and degree granting agencies are encouraged to think about resource allocation beyond the silos of individual courses and departments in order to sustain interdisciplinary teaching and learning frameworks like EDUCE.

## Supporting information

S1 Text EDUCE course surveys. (A) Fall 2017 pre-course survey. (B) 2018–2019 pre-course survey. (C) Fall 2017 post-course survey. (D) 2018–2019 post-course survey. In 2018, surveys transitioned from FluidSurveys to Qualtrics per UBC’s survey tool usage guidelines. EDUCE, Experiential Data science for Undergraduate Cross-Disciplinary Education; UBC, University of British Columbia.
(PDF)
